# 

**Published:** 1794

**Authors:** 


### MEDICAL FACTS

AND

### OBSERVATIONS.

VOL. V.

